# Preparation and Application of Water-in-Oil Emulsions Stabilized by Modified Graphene Oxide

**DOI:** 10.3390/ma9090731

**Published:** 2016-08-26

**Authors:** Xiaoma Fei, Lei Xia, Mingqing Chen, Wei Wei, Jing Luo, Xiaoya Liu

**Affiliations:** The Key Laboratory of Food Colloids and Biotechnology, Ministry of Education, School of Chemical and Material Engineering, Jiangnan University, Wuxi 214122, China; feixiaoma24@163.com (X.F.); sunnyskyxl@163.com (L.X.); wwei1985@jiangnan.edu.cn (W.W.); lxy@jiangnan.edu (X.L.)

**Keywords:** modified graphene oxide, Pickering emulsion, water-in-oil emulsions, nanocomposite

## Abstract

A series of alkyl chain modified graphene oxides (AmGO) with different alkyl chain length and content was fabricated using a reducing reaction between graphene oxide (GO) and alkyl amine. Then AmGO was used as a graphene-based particle emulsifier to stabilize Pickering emulsion. Compared with the emulsion stabilized by GO, which was oil-in-water type, all the emulsions stabilized by AmGO were water-in-oil type. The effects of alkyl chain length and alkyl chain content on the emulsion properties of AmGO were investigated. The emulsions stabilized by AmGO showed good stability within a wide range of pH (from pH = 1 to pH = 13) and salt concentrations (from 0.1 to 1000 mM). In addition, the application of water-in-oil emulsions stabilized by AmGO was investigated. AmGO/polyaniline nanocomposite (AmGO/PANi) was prepared through an emulsion approach, and its supercapacitor performance was investigated. This research broadens the application of AmGO as a water-in-oil type emulsion stabilizer and in preparing graphene-based functional materials.

## 1. Introduction

Owing to its water dispersibility, graphene oxide (GO), as a derivative of graphene, has many applications such as transparent conductors, sensors, polymer composites and energy storage [1,2,3,4,5,6,7]. GO is typically synthesized by chemical oxidation and exfoliation of graphite powders using strong oxidizing agents. After oxidation, the GO sheets are derivatized by a 2D lattice of partially broken sp^2^-bonded carbon networks with phenol, hydroxyl, and epoxide groups on the basal planes and carboxylic acid groups at the edges. Such a structure makes GO an amphiphile with a large hydrophobic basal plane and hydrophilic edges [8,9,10,11].

Since the pioneering works of Huang devoted to investigated the interfacial activity of GO at air-water, liquid-liquid, and liquid-solid interfaces and proved GO can act as a colloidal surfactant, many interesting works in the last several years have been focused on making GO at the water/oil interface [12,13,14,15,16,17,18,19]. He et al. investigated extensively the effects of different conditions such as type of oil, the sonication time, the GO concentration, the oil/water ratio, and the pH value on the properties of the Pickering emulsions stabilized by GO [16]. Creighton et al., presented the thermodynamic analysis of the behavior of 2D materials such as GO at liquid−liquid interfaces with applications in emulsification [17]. McCoy et al. demonstrated that oil/water emulsions stabilized by GO could be flocculated by either an increase or a decrease in pH. Such a flocculation was fully reversible at highly acidic pH and irreversible at high pH [18]. More than adsorption to the oil/water interface, Sun and co-workers reported that the surfactancy of GO can be enhanced by a block copolymer as a ligand. They added the copolymer ligand into oil, so GO can be trapped at water/oil interface and jammed into a solid thin film. Its kinetics were studied, but the emulsion properties of GO were not investigated and application of this GO thin film was not very extensive [19].

Making GO at the interface could provide guidance for the fabrication of graphene-based functional materials with specific structure and performance. Because the Pickering emulsions stabilized by GO can be used as a soft template to design new functional hybrid materials [20,21,22,23,24,25,26]. It should be pointed out that the graphene-based composites were usually synthesized through an “emulsion polymerization” or “suspension polymerization” since the graphene moieties very could well stabilize the monomer. Xie et al. reported the preparation of polystyrene (PS) particles via Pickering emulsion polymerization using GO as the stabilizer [27]. A similar “Pickering emulsion polymerization” approach was also reported by Kattimuttathu and co-workers; they used a so-called “seeded” emulsion polymerization to developed GO/PS nanocomposites [28]. Dao and co-workers prepared a microsphere of poly (methyl methacrylate) (PMMA)/graphene composite with a core–shell structure by Pickering suspension polymerization. First, they used GO to stabilize MMA (methylmethacrylate) in water, and then activated the incorporated initiator, AIBN, to turn the emulsified droplets into solid PMMA beads [29].

As stated above, Pickering emulsion stabilized by GO tends to be oil-in-water (O/W) type, which is not suitable for applications where the water-in-oil (W/O) type emulsion is needed. Therefore, some research has been focusing on the preparation of graphene-based W/O emulsion [25,30,31,32,33]. Woltornist and co-workers demonstrated the use of graphene/graphite to stabilize water in styrene/divinylbenzene and then curing the continuous monomer phase. After evaporation of the water, a semi-open cell carbon (graphene) network with high electrical conductivities was produced [32]. Zheng and co-workers reported when GO was modified by the cationic surfactant cetyltrimethylammonium bromide (CTAB), it could stabilize a W/O Pickering emulsion. They used the Pickering high internal phase emulsion stabilized by GO as templates and successfully prepared macroporous polymer/GO composites with a high specific surface area of about 490 m^2^·g^−1^ [25].

In this paper, a series of alkyl chain modified GO (AmGO) was synthesized, and then they were served as efficient W/O type emulsion stabilizers. The effects of alkyl chain length and alkyl chain content on the emulsion properties of AmGO were studied. Then the application of W/O emulsion stabilized by AmGO was investigated. The emulsion stabilized by AmGO was used as a soft template to prepared AmGO/polyaniline nanocomposite (AmGO/PANi). The AmGO/PANi exhibited a different nanostructure and a better supercapacitor performance than the common graphene oxide/polyaniline nanocomposite. 

## 2. Experimental Materials

Natural flake graphite was obtained from Qingdao Zhongtian Co., Ltd. (Qingdao, China) with a particle size of 400 meshes. All other chemicals are of analytical grade and used as received without further purification. Deionized (DI) water was used for the washings.

### 2.1. Preparation of Graphene Oxide (GO)

GO was prepared with the Hummers method and purified. Graphite (5 g) and NaNO_3_ (2.5 g) were mixed with 120 mL of H_2_SO_4_ (95%) in a 500 mL flask. The mixture was stirred for 30 min within an ice bath. While maintaining vigorous stirring, potassium permanganate (15 g) was added to the suspension. The rate of addition was carefully controlled to keep the reaction temperature lower than 20 °C. The ice bath was then removed, and the mixture was stirred at room temperature overnight. As the reaction progressed, the mixture gradually became pasty, and the color turned into light brownish. At the end, 150 mL of H_2_O was slowly added to the pasty with vigorous agitation. The reaction temperature was rapidly increased to 98 °C with effervescence, and the color changed to yellow. The diluted suspension was stirred at 98 °C for one day. Then, 50 mL of 30% H_2_O_2_ was added to the mixture. For purification, the mixture was washed by rinsing and centrifugation with 5% HCl and water for several times. Then, GO was dried under vacuum and obtained as a gray powder.

### 2.2. Preparation of Alkyl Chain Modified Graphene Oxide (AmGO)

GO (0.3 g) was dispersed and exfoliated in 150 mL deionized water via ultrasonication. The resulting suspension was mixed with the solution with a certain amount of alkyl amine (n-hexylamine, dodecylamine or octadecylamine) in 50 mL ethanol in a three-neck flask. The mixture was refluxed with mechanical stirring for 20 h at 90 °C. The precipitate was filtrated with a PP membrane with an average pore size of 220 nm. The filtrated powder was redispersed in 50 mL ethanol via ultrasonication for 2 min and then refiltrated. The rinsing-filtration process was repeated for 4 times to remove the physically absorbed alkyl amine. Finally, the mixture was dried at 60 °C under vacuum. 

### 2.3. Preparation of Pickering Emulsions Stabilized by Amgo

AmGO was dispersed in toluene via ultrasonication. The resulting dispersion was mixed with a certain amount of deionized water at different pH values or salt concentrations in glass vessels at room temperature. Then the mixtures were homogenized at 3000 rpm for 1 min by a XHF-DH-speed dispersator homogenizer (1 cm head) (Scientz, Ningbo, China). The type of emulsions was determined by drop test.

### 2.4. Preparation of Graphene/Polyaniline Nanocomposite

For the synthesis of AmGO/PANi, AmGO and aniline were dispersed in toluene via ultrasonication in glass vessels at room temperature. 10 mL of such dispersion was mixed with 1 mL of 1 M HCl contain a certain amount of APS. The mixture was homogenized at 3000 rpm for 1 min and then reacted at 0 °C. After reaction, the product was vacuum filtered and washed with acetone and deionized water to remove excess acid, possible oligomers. The obtained product was dried completely at 50 °C. For the synthesis of GO/PANi, aniline and GO were dispersed in water via ultrasonication. Then contain a certain amount of HCl solution and APS were added into the mixture. The mixture was reacted at 0 °C. After reaction, the product was vacuum filtered and washed with acetone and deionized water.

### 2.5. Characterization

The photos of the emulsions stabilized by alkyl chain modified GO were recorded with a digital camera (COOLPIX P100, Nikon, Tokyo, Japan). The optical micrographs of the prepared emulsions on transparent glass slides were taken using an optical microscope (DM-BA450, Motic China Group Co., Ltd., Xiamen, China). The average sizes and size distributions of the emulsion droplets were determined based on the images of the emulsion droplets using Nano Measurer 1.2 software. The volume fraction of the stable emulsion was calculated by dividing the height of the emulsion phase by the total height of the initial emulsion. Water contact angle measurements were performed at ambient conditions using an OCA15EC goniometer with a charge-coupled device camera equipped for image capture (Dataphysics, Beijing, China). The GO and AmGO powder samples were first made into a film by a tableting machine. Typically, 0.5 g sample was used and the pressure was kept at 15 MPa for 2 min. The obtained film was around 0.1 mm thick. Static contact angle measurements were performed by placing a 2 μL deionized water droplet on the surface of the prepared film. The axisymmetric-drop-shape analysis profile (ADSA-P) method was used for estimating the contact angle of water on the film surface. The SEM image was taken in an S-4800 field emission scanning electron microscope (Hitachi, Tokyo, Japan). Raman spectra were recorded using a Renishaw in Via Raman Microscope (Renishaw, Gloucestershire, UK) operating at 785 nm with a charge-coupled device detector. FT-IR spectroscopy were collected on a Nicolet IS-50 FT-IR spectrometer equipped with a Smart OMNI sampler with a high purity Ge crystal and diamond crystal (Nicolet, Green Bay, WI, USA). Electrochemical characterization was carried out in a conventional three-electrode cell system with a nanocomposite modified glassy carbon electrode (GCE) was used as the working electrode. First, GCE was carefully polished with alumina powders (0.3 and 0.05 mm) on a polishing cloth, then sonicated in ethanol and dried at room temperature. To prepare working electrode, typically, 50 mg of nanocomposite was homogeneously dispersed in 50 mL of water under ultrasonication to obtain the dispersion with concentration of 1.0 mg·mL^−1^. Then 10 μL of the obtained dispersion was dropped onto the GCE surface, and then dried at room temperature to form the nanocomposite modified GCE. Electrochemical measurement was performed on a CHI660e electrochemical workstation (Shanghai Chenghua Instrument Company, Shanghai, China) in a conventional three-electrode cell system. The nanocomposite modified GCE, a saturated calomel electrode (SCE) and a Pt wire were used as working electrode, reference electrode and counter electrode, respectively. Galvanostatic charge/discharge curve was measured in a voltage from 0 to 0.8 V with 1.0 M H_2_SO_4_ as the electrolyte. 

## 3. Results and Discussion

### 3.1. Basic Characterization of AmGO

The GO used in this work was prepared from purified natural graphite via a modified Hummers method [34]. The AmGO was synthesized using different alkyl amine to reduce GO according to previous report [35,36]. Here, three alkyl amine with different alkyl chain length (n-hexylamine (6-C), dodecylamine (12-C) or octadecylamine (18-C)) were utilized to modified GO. AmGO with different GO and octadecylamine ratios were also synthesised. For easy expression, the alkyl chain length in AmGO was labeled as X, and the weight ratio between GO and alkyl amine in AmGO was labeled as Y. Thus, samples with different alkyl chain length and alkyl chain content were labeled as AmGOX-Y. For example, AmGO18-1 represented for the octadecylamine (18-C) modified GO with the weight ratio of 1:1.

**Figure 1:**
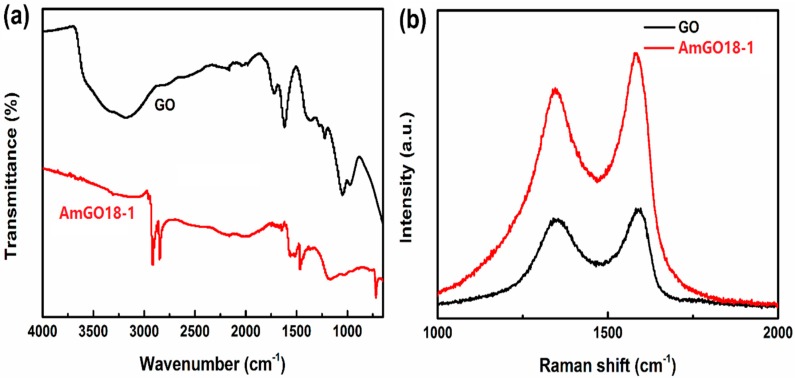
(**a**) FTIR spectra of GO and AmGO18-1; (**b**) Raman spectra of GO and AmGO18-1.

The basic characterization is given in Figure 1. Figure 1a shows the FTIR spectra of GO and AmGO18-1. After modified with alkyl amine, the bands of GO at 1725 cm^−1^ and 1581 cm^−1^ which should be assigned to the C=O carboxyl stretching vibration band and C=C in aromatic ring were disappeared. A new band at 1541–1603 cm^−1^ assigned to the carboxylic acid salt (COO^−^) asymmetric stretch mode was observed. Peaks at 2918, 2848 and 720 cm^−1^ are due to the –CH_2_– in alkyl chain in the spectrum of AmGO18-1. The result of Raman was given in Figure 1b. Both the two samples showed two bands in Raman spectra, D band and G band, which suggested that the skeleton structure of GO remained in AmGO18-1 after modification. However, after modification, the I_D_/I_G_ value decreased, which means GO was reduced in this process. This result is in consistent with previous literatures, which were also modifying the GO with alkyl amine and proved GO could be reduced [32,35]. Both the FTIR and Raman results demonstrate the successful preparation of AmGO.

### 3.2. Effect of Alkyl Chain Length on the Emulsion Properties of AmGO

**Figure 2:**
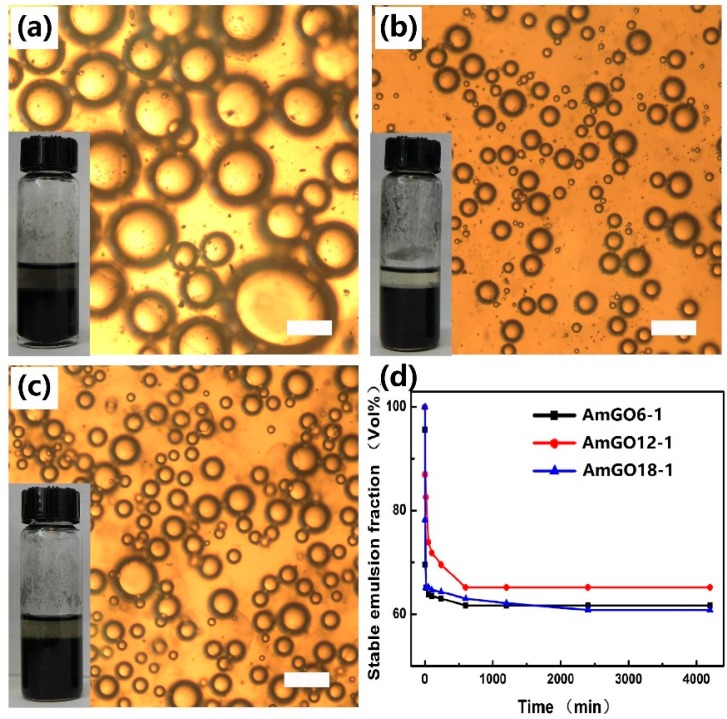
Optical micrographs and photographs 72 h after preparation of water-in-toluene Pickering emulsions stabilized by GO modified with different organic amine: (**a**) AmGO6-1; (**b**) AmGO12-1 and (**c**) AmGO18-1; (**d**) Change in the volume fraction of the residual emulsion as a function of time for the emulsions stabilized by GO modified with different organic amine. Concentration: 1 mg/mL. Toluene/water ratio: 1:1.

**Figure 3:**
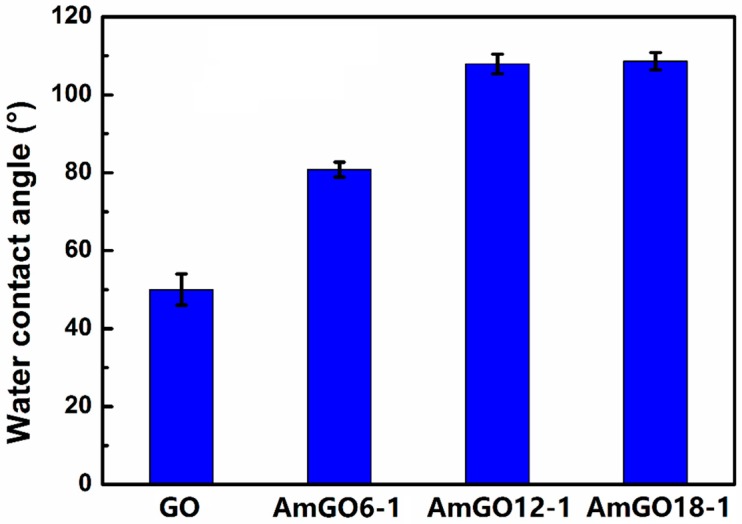
Water contact angles of GO, AmGO6-1, AmGO12-1 and AmGO18-1.

As is known, GO is highly hydrophilic owning to the oxygen-containing functional groups, such as phenol, hydroxyl, epoxide and especially the edge carboxylic acid groups. So GO tends to stabilize O/W type emulsion. In most cases, the emulsion properties of a stabilizer is largely related to its amphipathicity. Here, different alkyl amines were utilized to introduce hydrophobic alkyl chains on GO surface to adjust its amphipathicity. As all the AmGO could disperse in toluene, toluene was chosen as oil phase to investigate the emulsion properties. First, the effect of alkyl chain length on the emulsion properties was investigated. The results were given in Figure 2. Clearly, all the three AmGO samples (AmGO6-1, AmGO12-1, AmGO18-1) were observed to efficiently stabilize emulsions from toluene/water mixtures. The emulsions stayed stable for at least several months but sedimented, leaving a clear oil phase above the emulsion. Such a result showed the good stability of emulsion stabilized by AmGO. Compared with the emulsion stabilized by GO, which is O/W type, all the emulsions stabilized by AmGO here belonged to W/O type. This could be explained by the following: it is well known that if a stabilizer is more soluble in the oil phase, it is easily to obtain a W/O emulsion. In this study, GO is more soluble in water, so it can emulsify toluene in water. For AmGO, after being modified with alkyl chain, the AmGO becomes more hydrophobic and could easily disperse in the toluene, which is the oil phase. Thus, AmGO tends to stabilize the W/O emulsion. The water contact angles were measured to illustrate such an increase in hydrophobicity (Figure 3). The water contact angle of GO was 50°, which is much smaller than 90°, showing that GO is quite hydrophilic and tend to obtain O/W type emulsion. For AmGO, the water contact angles were much higher than that of pure GO and increased with raising the length of alkyl chain. What should be pointed out is that the water contact angle of AmGO6-1 was 81°, which is still less than 90°. As the length of the alkyl chain increases, both AmGO12-1 and AmGO18-1 showed water contact angles higher than 90° and possessed enough hydrophobicity, leading to good emulsion properties. In addition, all the emulsions stabilized by AmGO reached a stable state very quickly and the volume fractions of the residual emulsion were more than 60% (Figure 2d). 

### 3.3. Effect of the Grafting Content of Alkyl Chain on the Emulsion Properties of AmGO

**Figure 4:**
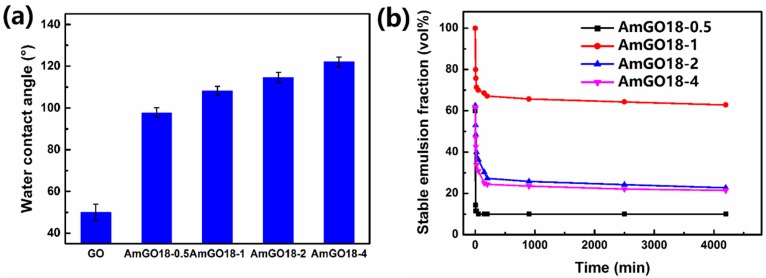
(**a**) Water contact angles of GO and AmGO; (**b**) Change in the volume fraction of the residual emulsion as a function of time for the emulsions stabilized by AmGO18-0.5, AmGO18-1, AmGO18-2 and AmGO18-4. AmGO concentration: 1 mg/mL. Toluene/water ratio: 1:1.

**Figure 5:**
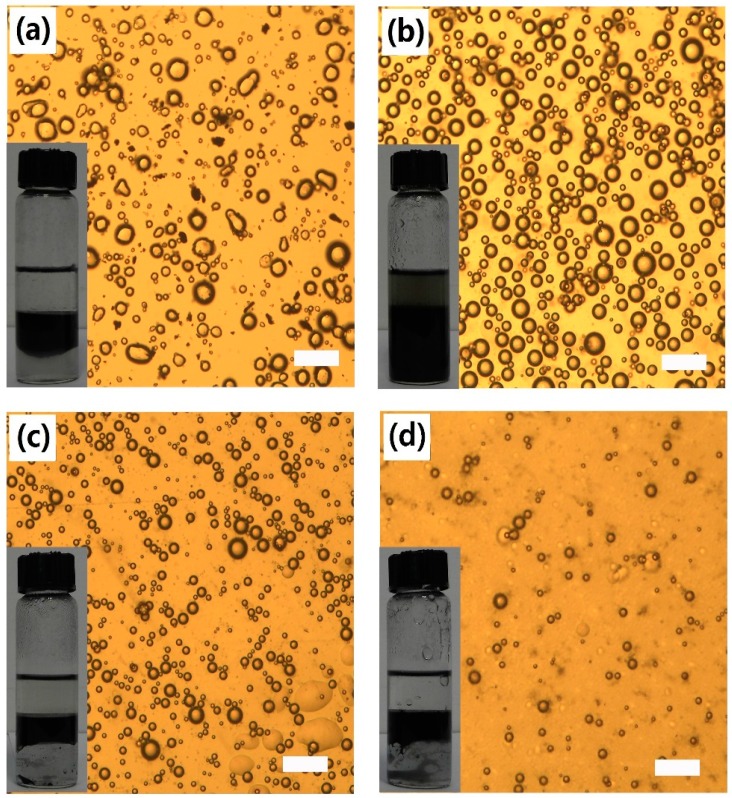
Optical micrographs and photographs after 72 h preparation of Pickering emulsions stabilized by AmGO: (**a**) AmGO18-0.5; (**b**) AmGO18-1; (**c**) AmGO18-2 and (**d**) AmGO18-4. AmGO concentration: 1 mg/mL. Toluene/water ratio: 1:1. Scale bar: 100 μm.

The amphipathicity of AmGO could also be tuned by the grafting content of alkyl chain. Here, four AmGO with different GO and octadecylamine ratios were synthesized to illustrate the effect of the grafting content on emulsion properties of AmGO. As expected, the hydrophobicity increased with increasing grafting content of octadecylamine. This could be witnessed by the results of water contact angles (Figure 4a). The water contact angles of all the AmGO were larger than 90° and increased with the content of octadecylamine. The emulsion properties of these four AmGO were given in Figure 5. All the four AmGO could form water/toluene type emulsions. Obviously, droplets of the emulsion stabilized by AmGO18-1 were very uniform and the stable emulsion fraction reached 65%, which was much higher than those of other three AmGO (Figure 4b). For the emulsion stabilized by AmGO18-0.5, the morphology of the emulsion droplet was irregular, and free AmGO18-0.5 sheets were observed. This could be explained by the fact that the content of octadecylamine on GO is too low, so AmGO18-0.5 could not disperse in toluene efficiently and settle down in the bottom, leading to poor emulsion properties. For the emulsions stabilized by AmGO18-2 and AmGO18-4, their emulsion droplet sizes were similar to that of AmGO18-1. However, the stable emulsion fractions were about 25%, which was much lower than that of AmGO18-1. The reason for this observation is that there is too many hydrophobic octadecylamine chains on GO surface for AmGO18-2 and AmGO18-4, which makes the materials too hydrophobic and thus decreases their active surface.

### 3.4. Effect of pH Value and NaCl Concentration on the Emulsion Properties of AmGO

**Figure 6:**
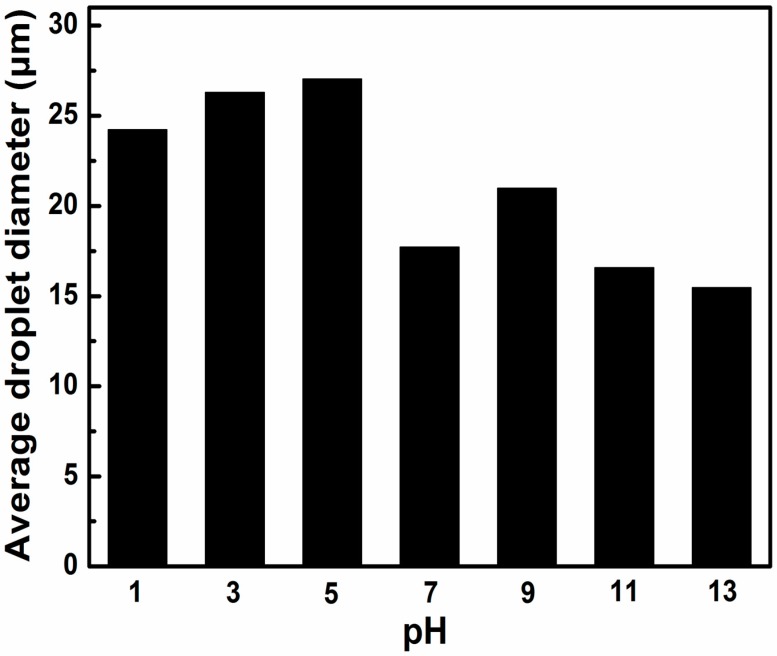
Average droplet diameter of Pickering emulsions stabilized by AmGO18-1 as a function of pH values. AmGO18-1 concentration: 1 mg/mL. Toluene/water ratio: 1:1. Scale bar: 100 μm.

The amphiphilicity of GO, as pointed out above, largely depends on the edge carboxyl groups. Thus, the emulsion properties of GO is strongly influenced by varying the pH values due to protonation and deprotonation of the edge carboxyl groups. McCoy reported the pH-dependent dispersion and flocculation of the emulsion stabilized by GO through either an increase or a decrease in pH [18]. Here the effect of pH value on the emulsion properties of AmGO18-1 was investigated, and the results are shown in Figure 6 and Figure S1. All the emulsions stabilized by AmGO18-1 at different pH values showed similar emulsion droplet size and similar stable emulsion volume. Such a result means that the emulsion properties of AmGO18-1 was not influenced by pH, which is in contrast with GO. This is because GO was partly reduced, so the oxygen-containing functional groups on GO decreased, which significantly decreased the sensitivity to pH value. However, it should be noted that the emulsion droplet size become smaller and more uniform and the toluene phase became transparent in basic conditions. This means that nearly all the AmGO18-1 in the dispersion was associated with the emulsification at high pH. 

It was reported that emulsion stabilized by GO was also affected by electrolytes, such as NaCl, which affect the aggregation behavior of the GO dispersion. This is because both the carboxylic acid and hydroxyl groups on GO surface are candidates for charge screening when the salt is added. For the emulsions stabilized by AmGO18-1, as expected, the effect of NaCl concentration on the emulsion droplet size and on the stable emulsion volume was not distinct. All the emulsions showed similar stable emulsion volumes and no flocculation was observed (Figure S2).

### 3.5. Effect of AmGO Concentration on the Emulsion Properties of AmGO

**Figure 7:**
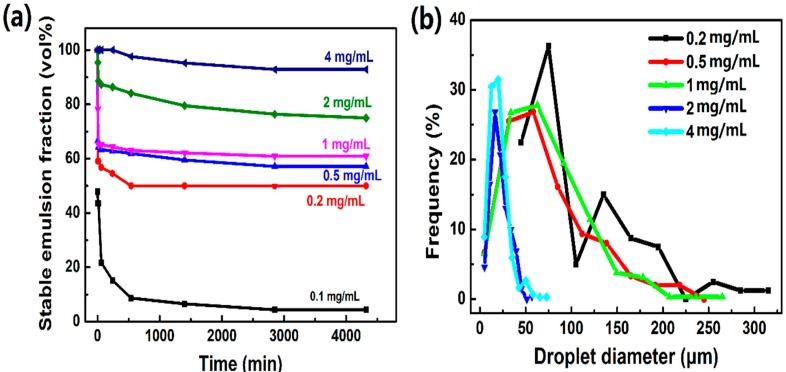
(**a**) Change in the volume fraction of the residual emulsion as a function of time for the emulsions stabilized by different concentrations of AmGO18-1; (**b**) Distribution of droplet diameters after the preparation of water-in-toluene emulsions at different AmGO18-1 concentrations.

Particle concentration is another important factor in the formation of Pickering emulsion, which has an influence on the emulsion stability and on the average droplet size. The microscopy images and distribution of droplet diameters for emulsions prepared with different AmGO12-1 concentrations were shown in Figure 7 and Figure S3. For AmGO18-1 concentration was 0.1 mg·mL^−1^, there was almost no emulsion formed. When the concentration of AmGO18-1 reached 0.2 mg·mL^−1^, a uniform W/O type emulsion was formed and there was no separated water phase. This obvious change could be explained as following. AmGO has a very large specific surface area. The increase of AmGO18-1 concentration within a certain range significantly results in an increase of the total surface area. Such an increase of the surface area of AmGO18-1 causes a reduction in the free energy and makes the system more stable, thus producing good emulsion properties. For concentrations greater than 0.2 mg·mL^−1^, as expected, the average droplet size decreased and the volume fraction of the residual emulsion increased with increasing AmGO18-1 concentration. 

### 3.6. Preparation of AmGO/PANi and Its Supercapacitor Performance 

**Figure 8:**
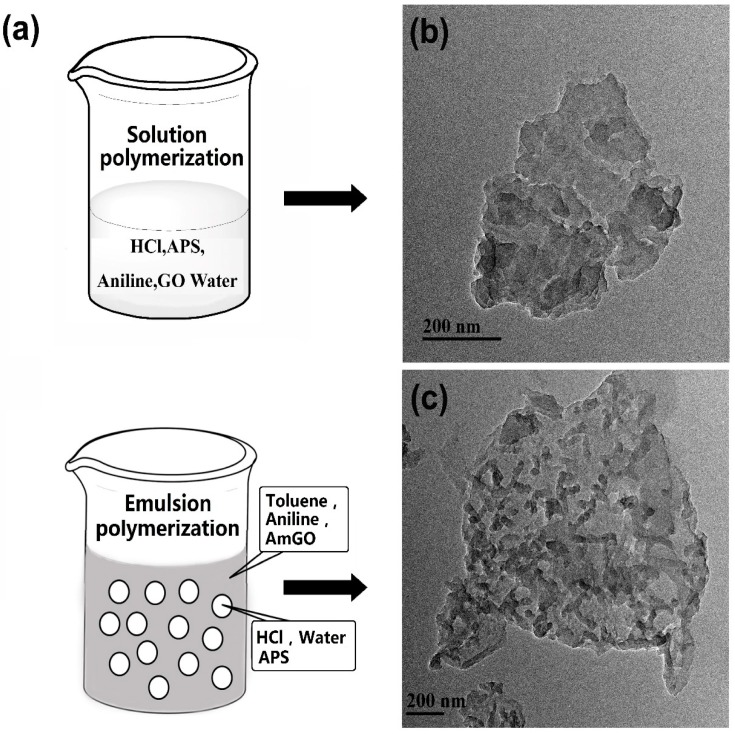
(**a**) Illustration of the solution polymerization and emulsion polymerization process; TEM images of (**b**) GO/PANi and (**c**) AmGO/PANi.

The main application of GO used as an emulsion stabilizer is to design novel functional graphene-based materials with specific structure and performance using emulsion stabilized by GO as a soft template. Much research has focused on preparing GO/PS material using emulsion polymerization [12,34,35]. However, as was mentioned above, emulsion stabilized by GO was highly influenced by pH value, which limited the choice of monomer, for example aniline. As we all know, polyaniline (PANi) is a typical conducting polymer which has been used in various fields [36,37,38]. Its synthesis is typically performed using oxidation polymerization in an acid environment. We had tried to prepare GO/PANi nanocomposite through emulsion polymerization using O/W emulsion stabilized by GO as a soft template (GO solution as water phase and toluene dissolved with aniline monomer as oil phase). The system could form O/W emulsion. However, after adjusting the pH value and adding APS to initiate polymerization, the reaction system flocculated rapidly, leaving brown precipitate in the bottle. This is because emulsion stabilized by GO is unstable under low pH values and became flocculate. As is demonstrated, emulsion stabilized by AmGO is very stable within a wide range of pH. Thus, AmGO/PANi nanocomposite was also prepared through emulsion polymerization using the AmGO18-1 as emulsion stabilizer (HCl solution dissolved with APS as water phase and toluene dispersed with AmGO18-1 and aniline monomer as oil phase). As expected, the reaction system remained stable during the whole polymerization progress. To understand the difference in structure between the graphene/polyaniline composite synthesized through the emulsion strategy and the common solution method, we also prepared GO/PANi nanocomposite using a common solution polymerization. It is easy to realize that for the GO/PANi, the polymerization of aniline followed a surface-initiated polymerization mechanism. Unlike GO/PANi, the obtained AmGO/PANi nanocomposite synthesised through an emulsion strategy showed an interfacial polymerization mechanism. Such a difference could be demonstrated by the results of TEM. It can be seen that the PANi nanofibers and nano-spheres were formed and randomly attached on the AmGO surface. The diameter of PANi nanofibers was around 39 nm [39,40]. However, the morphology of GO/PANi was very different from that of AmGO/PANi, i.e., the GO sheet were homogeneously covered by PANi layer and no specific nanostructure was observed (Figure 8b). This revealed a surface-initiated polymerization process. 

**Figure 9:**
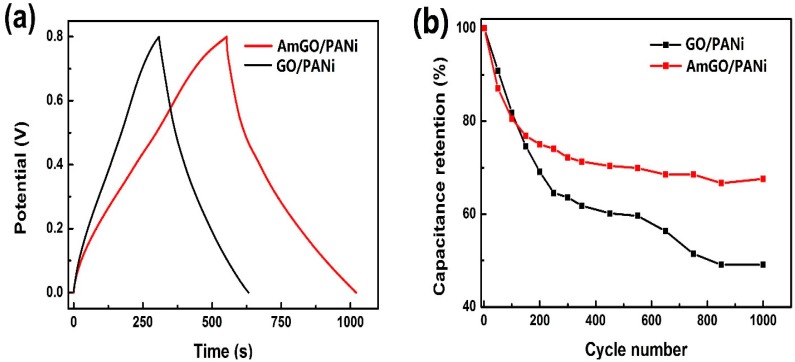
(**a**) charge-discharge curves of AmGO/PANi and GO/PANi at a current density of 1 A·g^−1^ and (**b**) cycling stability of AmGO/PANi and GO/PANi at a current density of 2 A·g^−1^.

The capacitive behavior difference between AmGO/PANi and GO/PANi was confirmed by galvanostatic charge/discharge curves and the curves at a current density of 1 A·g^−1^ are shown in Figure 9a. Apparently, the AmGO/PANi prepared from emulsion polymerization method exhibited a better supercapacitor performance than GO/PANi. The specific capacitance of AmGO/PANi reached 585 F·g^−1^ at a current density of 1 A·g^−1^, which was much higher than that of GO/PANi (405 F·g^−1^). The electrochemical stability of AmGO/PANi and GO/PANi nanocomposites were also evaluated by consecutive charge/discharge cycles at a current density of 2 A·g^−1^. As shown in Figure 9b, the AmGO/PANi exhibited a better cycling stability than GO/PANi. About 67.8% of the initial capacitance could be maintained after 1000 cycles at a current density of 2 A·g^−1^, much higher than that of GO/PANI (49%). Such a better specific capacitance and cycling life may be due to the special structure in the nanocomposite obtained through emulsion polymerization method using the AmGO as W/O emulsifier.

## 4. Conclusions

In conclusion, we have shown the preparation and application of W/O type emulsions stabilized by AmGO. The effects of alkyl chain length and alkyl chain content on the emulsion properties of AmGO were investigated. The results showed that the emulsion properties of AmGO significantly depend on its amphipathicity. AmGO18-1 exhibited the best emulsion properties with a good emulsion stability within a wide range of pH (from pH = 1 to pH = 13) and salt concentrations (from 0.1 to 1000 mM). AmGO/PANi was synthesized using an emulsion approach. Compared with GO/PANi, AmGO/PANi exhibited a different nanostructure and a better supercapacitor performance. The specific capacitance of AmGO/PANi reached 585 F·g^−1^ at a current density of 1 A·g^−1^. This research broadens the application of AmGO as a W/O type emulsion stabilizer and in preparing graphene-based functional materials.

## Supplementary Materials

The following are available online at www.mdpi.com/1996-1944/9/9/731/s1. Figure S1: Optical micrographs and photographs after 72 h preparation of Pickering emulsions stabilized by AmGO18-1 at different pH values: (a) pH = 1; (b) pH = 3; (c) pH =5; (d) pH = 7; (e) pH = 9; (f) pH = 11 and (g) pH = 13. AmGO18-1 concentration: 1 mg/mL. Toluene/water ratio: 1:1. Scale bar: 100 μm; Figure S2: Photographs 72 h after preparation of Pickering emulsions stabilized by AmGO18-1 with different NaCl concentrations. The concentrations of NaCl (mM) are (a) 0.1; (b) 1; (c) 10; (d) 20; (e) 50; (f) 100; (g) 300; (h) 500; and (i) 1000. AmGO18-1 concentration: 1 mg/mL. Toluene/water ratio: 1:1; Figure S3: Optical micrographs and photographs after 72 h preparation of Pickering emulsions stabilized by AmGO18-1 at different AmGO18-1 concentrations: (a) 0.1; (b) 0.2; (c) 0.5; (d) 1; (e) 2; (f) 4 mg/mL. Toluene/water ratio: 1:1. Scale bar: 100 μm.
