# Supplementary material for: Preparation and Application of Water-in-Oil Emulsions Stabilized by Modified Graphene Oxide
Source: Materials (Basel). 2016 Aug 26;9(9):731. doi: 10.3390/ma9090731 (PMC5457042; doi:10.3390/ma9090731)
Supplement: Supplementary file 1 [file materials-09-00731-s001.pdf]

Supplementary Materials: Preparation and Application of Water-in-Oil Emulsions Stabilized by Modified Graphene Oxide

Xiaoma Fei, Lei Xia, Mingqing Chen, Wei Wei, Jing Luo and Xiaoya Liu

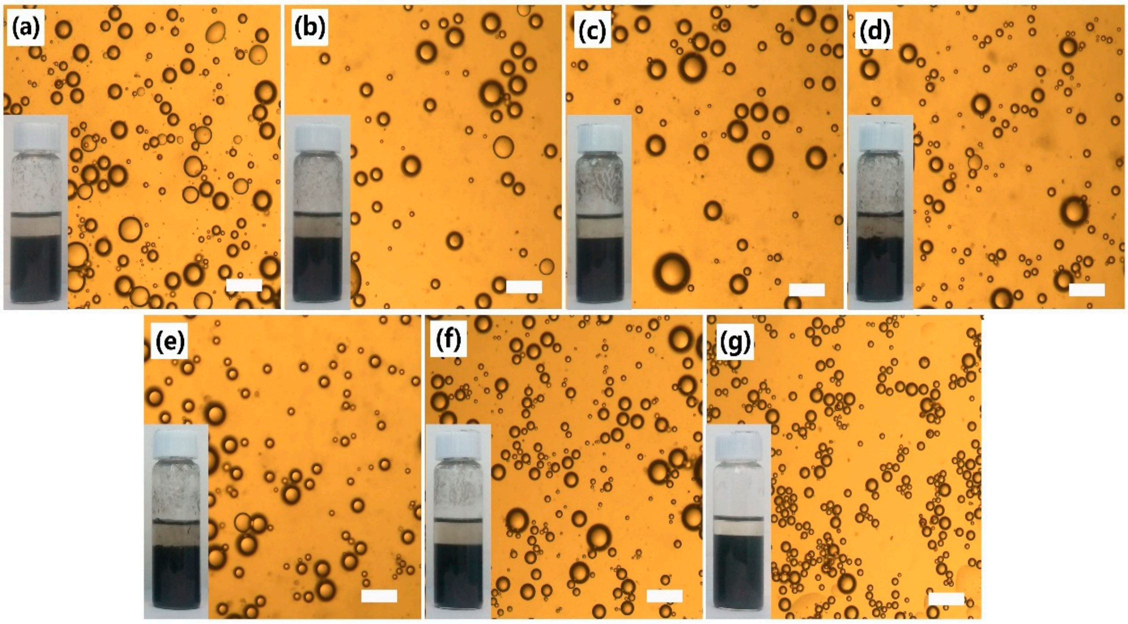

Figure S1. Optical micrographs and photographs after 72 h preparation of pickering emulsions stabilized by AmGO18-1 at different pH values: (**a**) pH = 1; (**b**) pH = 3; (**c**) pH = 5; (**d**) pH = 7; (**e**) pH = 9; (**f**) pH = 11 and (**g**) pH = 13. AmGO18-1 concentration: 1 mg/mL. Toluene/water ratio: 1:1. Scale bar: 100 μm.

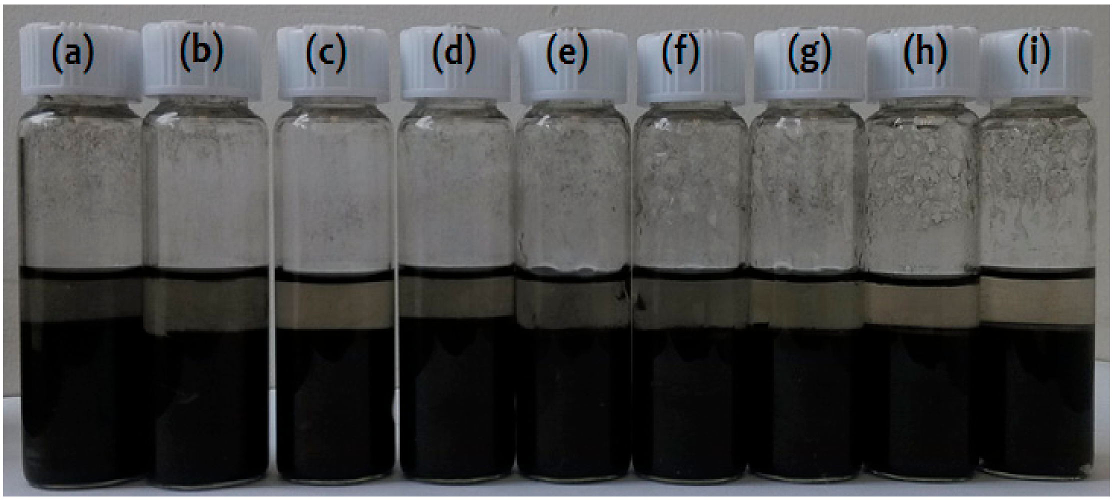

Figure S2. Photographs 72 h after preparation of pickering emulsions stabilized by AmGO18-1 with different NaCl concentrations. The concentrations of NaCl (mM) are (**a**) 0.1; (**b**) 1; (**c**) 10; (**d**) 20; (**e**) 50; (**f**) 100; (**g**) 300; (**h**) 500; and (**i**) 1000. AmGO18-1 concentration: 1 mg/mL. Toluene/water ratio: 1:1.

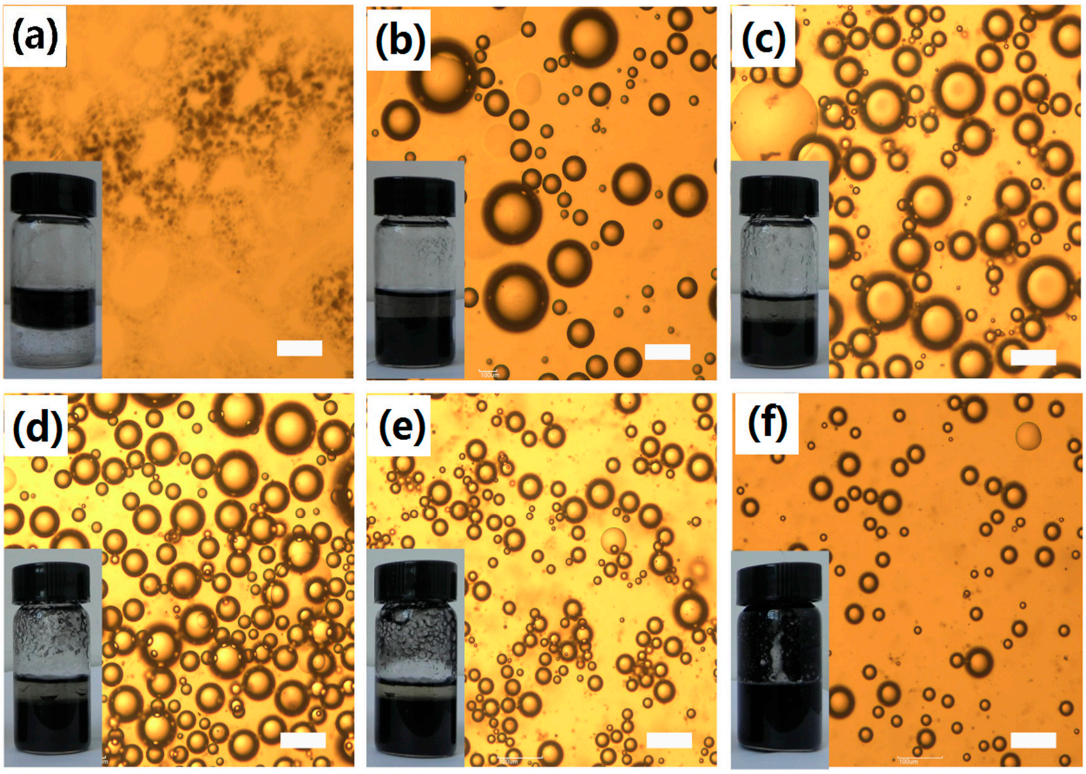

Figure S3. Optical micrographs and photographs after 72 h preparation of pickering emulsions stabilized by AmGO18-1 at different AmGO18-1 concentrations: (**a**) 0.1; (**b**) 0.2; (**c**) 0.5; (**d**) 1; (**e**) 2; (**f**) 4 mg/mL. Toluene/water ratio: 1:1. Scale bar: 100 μm.
